# *Symbiodinium* Photosynthesis in Caribbean Octocorals

**DOI:** 10.1371/journal.pone.0106419

**Published:** 2014-09-05

**Authors:** Blake D. Ramsby, Kartick P. Shirur, Roberto Iglesias-Prieto, Tamar L. Goulet

**Affiliations:** 1 Department of Biology, University of Mississippi, University, Mississippi, United States of America; 2 Unidad Académica de Sistemas Arrecifales (Puerto Morelos), Instituto de Ciencias del Mar y Limnología, Universidad Nacional Autónoma de México, Cancún, México; University of Hyderabad, India

## Abstract

Symbioses with the dinoflagellate Symbiodinium form the foundation of tropical coral reef communities. Symbiodinium photosynthesis fuels the growth of an array of marine invertebrates, including cnidarians such as scleractinian corals and octocorals (e.g., gorgonian and soft corals). Studies examining the symbioses between Caribbean gorgonian corals and Symbiodinium are sparse, even though gorgonian corals blanket the landscape of Caribbean coral reefs. The objective of this study was to compare photosynthetic characteristics of Symbiodinium in four common Caribbean gorgonian species: Pterogorgia anceps, Eunicea tourneforti, Pseudoplexaura porosa, and Pseudoplexaura wagenaari. *Symbiodinium* associated with these four species exhibited differences in Symbiodinium density, chlorophyll a per cell, light absorption by chlorophyll a, and rates of photosynthetic oxygen production. The two Pseudoplexaura species had higher Symbiodinium densities and chlorophyll a per Symbiodinium cell but lower chlorophyll a specific absorption compared to P. anceps and E. tourneforti. Consequently, P. porosa and P. wagenaari had the highest average photosynthetic rates per cm^2^ but the lowest average photosynthetic rates per Symbiodinium cell or chlorophyll a. With the exception of Symbiodinium from E. tourneforti, isolated Symbiodinium did not photosynthesize at the same rate as Symbiodinium in hospite. Differences in *Symbiodinium* photosynthetic performance could not be attributed to *Symbiodinium* type. All *P. anceps* (n = 9) and *P. wagenaari* (n = 6) colonies, in addition to one *E. tourneforti* and three *P. porosa* colonies, associated with *Symbiodinium* type B1. The B1 *Symbiodinium* from these four gorgonian species did not cluster with lineages of B1 *Symbiodinium* from scleractinian corals. The remaining eight *E. tourneforti* colonies harbored *Symbiodinium* type B1L, while six *P. porosa* colonies harbored type B1i. Understanding the symbioses between gorgonian corals and *Symbiodinium* will aid in deciphering why gorgonian corals dominate many Caribbean reefs.

## Introduction

Gorgonian corals (subclass Octocorallia) are abundant and important members of coral reef communities throughout the Caribbean [1]–[7]. Unlike the dramatic decline of scleractinian corals in the Caribbean [8]–[11], gorgonian coral abundance is steady or even increasing [7], [12], [13]. For example, in the Florida Keys, gorgonian octocoral abundance increased significantly since 1999 [7]. And, in the US Virgin Islands, the abundance of two of three gorgonian species studied has increased since 1992 [13]. Gorgonian corals are also abundant in the Yucatan coast of México, where gorgonian species richness can exceed scleractinian coral species richness [14].

Despite being prominent members of Caribbean reef communities, studies on gorgonian coral physiology are sparse, predominantly focusing on the gorgonian coral hosts without addressing their symbiosis with the unicellular dinoflagellates in the genus *Symbiodinium*. Studies tracked digested material [15], [16], described sclerite (microscopic skeletal elements) formation [17], [18], or measured growth [19], [20], and feeding rates [21], [22]. In addition, gorgonian secondary metabolites have been extensively studied due to their medical and economic importance [23]. Two early studies measured the photosynthetic rates of *Symbiodinium* in a few Caribbean gorgonian species [24], [25], while key photosynthetic characteristics, including light absorption efficiencies, have not been measured. Furthermore, the handful of studies that investigated the physiology of Caribbean gorgonian corals and their Symbiodinium [24]–[29] did not identify the *Symbiodinium* present.

*Symbiodinium* are currently divided into nine phylogenetic clades, A-I [30], although clade E may represent a single species [31]. Within each clade, *Symbiodinium* are often distinguished using sequences of the internal transcribed spacer regions of ribosomal DNA (*Symbiodinium* types sensu [32]). Almost all Caribbean gorgonian species associate with *Symbiodinium* clade B types and many harbor type B1 [33], [34]. Within type B1, multiple lineages have been identified [35], [36]. Hosting different *Symbiodinium* types can correlate with ecological [36], [37] and physiological differences [38] between cnidarian hosts. Often, however, physiological differences are assessed when the cnidarians face stressful environmental conditions [38].

Collecting baseline physiological data on coral-algal symbioses [39], and not just data when the symbioses are stressed, are critical to evaluating the effects of stressors on symbioses [40]. The objective of this study was to characterize the photosynthesis of Symbiodinium, in hospite and in isolation, in four common Caribbean gorgonian species: *Pterogorgia anceps*, Eunicea tourneforti, *Pseudoplexaura porosa*, and *Pseudoplexaura wagenaari*. Studying the physiology of the symbiosis between gorgonian corals and *Symbiodinium* may shed light on why gorgonian corals dominate Caribbean reefs while scleractinian coral abundance is declining.

## Materials and Methods

### Sample collection and acclimation

In June 2010, gorgonian branches were collected at 3 m depth from a patch reef near Puerto Morelos, Quintana Roo, México (20° 52' N, 86° 51' W, permit number DGOPA.11519,121109.3949, Secretaria de Medio Ambiente y Recursos Naturales, México). One branch was removed from each sampled colony of the four gorgonian species: Pterogorgia anceps (n = 9), Eunicea tourneforti (n = 9), Pseudoplexaura porosa (n = 9), and Pseudoplexaura wagenaari (n = 6). In order to maintain their natural orientation, the branches were attached to vertical PVC stands and were held in outdoor aquaria with flowing seawater for 11 days for acclimation. The temperature in the aquaria was determined using Hobo pendant data loggers (Onset Computer Corporation, MA, USA), and it was maintained at 29.5±0.5°C, similar to the ambient temperature on the reef, by using an aquarium chiller (0.5hp Delta Star, Aqua Logic, USA) and heaters (1000 W and 1800 W EasyPlug heater, Process Technologies, USA). Light levels in the aquaria were maintained at levels similar to those at the collection site (∼900 µmol quanta m^−2^ s^−1^ at solar noon) by shading the aquaria with window screening.

### Photochemical efficiency of photosystem II in Symbiodinium

Throughout the acclimation period, the photochemical efficiency of photosystem II of the Symbiodinium in each branch was measured using a diving pulse amplitude modulated (PAM) fluorometer (WALZ, Effeltrich, Germany) at solar noon (Δ*F/F_m_'*; effective yield) and after sunset (*F_v_/F_m_*; maximum yield). Photochemical efficiency was measured approximately 2 cm below the branch tip, on the side of the branch, at a constant distance from the surface of the branch. Using the effective and maximum yield values obtained for each species, we calculated the maximum excitation pressure over photosystem II, *Q_m_,* whereby *Q_m_* = 1-((Δ*F/F_m_'*)/(*F_v_/F_m_*)) [41].

### Oxygen flux of *Symbiodinium* in gorgonian branches

After 11 days of acclimation, the oxygen fluxes of the branches were measured using Clark-type oxygen electrodes in twin 0.5 l acrylic chambers. The electrodes were connected to a 782 Oxygen Meter (Strathkelvin Instruments Ltd., North Lanarkshire, Scotland). The chambers were filled with 0.45 µm filtered seawater with 4 mM sodium bicarbonate [42]. Water was circulated inside each chamber using a small pump. Electrodes were calibrated by bubbling oxygen and nitrogen gas to define 100% and 0% oxygen concentrations, respectively. A water jacket, connected to a water recirculator, maintained the temperature of 29.0–29.5°C inside the chambers.

To measure the oxygen flux of a gorgonian branch, a branch was sealed in a chamber. Following a 20 min acclimation, respiration was measured for 10 min in darkness. Then, the branch was illuminated from one direction with three 6 W LED light bulbs, while white acrylic on the sides and rear of the chamber reflected the light within the chamber. The branch was exposed to 11 irradiance levels (0–2200 µmol quanta m^−2^ s^−1^) by progressively removing sheets of window screening. The irradiance levels in each chamber were measured using a 4π quantum sensor (WALZ, Effeltrich, Germany). A gorgonian branch was exposed to each irradiance level for up to 15 min, or until a linear change in oxygen concentration was recorded in the chamber. Following the last light level, respiration was measured again in darkness.

Oxygen flux measurements required 2.5 h per sample and were collected at approximately 09:00 or 12:00 local time. Due to the lengthy data collection time, it took 10 days to measure the oxygen fluxes of all the gorgonian branches. To account for changes in photosynthetic rate at different times of day, the photosynthesis of Symbiodinium in each gorgonian species was measured at alternate times on consecutive days. We then used the oxygen flux data to produce photosynthesis versus irradiance (P-E) graphs (see below).

After measuring the oxygen flux, the surface areas of the gorgonian branches were calculated. For gorgonian species with cylindrical branches (E. tourneforti, P. porosa, and P. wagenaari), we measured the length and diameter of each branch and calculated the surface area of a cylinder. For P. anceps, whose branches are blade like, we measured the diameter and length of the blade and calculated the surface area by combining the surface area of all rectangular sides.

### Isolation and oxygen flux of isolated *Symbiodinium*

Branches were returned to shaded aquaria for 1 h before Symbiodinium isolation. *Symbiodinium* were isolated by homogenizing, in a mortar and pestle, a 2 cm section of the gorgonian branch, obtained 3 cm below the tip of the branch. The homogenate was diluted in 20 ml of 0.45 µm filtered seawater and centrifuged for 1 min at 500 rpm. The liquid fraction was filtered through 150, 74, and 20 µm nitex meshes. The filtered fraction was then centrifuged for 1 min at 3500 rpm and the Symbiodinium pellet was washed two times with 10 ml of filtered seawater. Following the last wash, the Symbiodinium pellet was resuspended in 5 ml of filtered seawater and divided into aliquots for oxygen flux measurements (0.5 ml), chlorophyll content (1.5 ml), cell density (0.25 ml), and genetic identification (2 ml).

To measure isolated Symbiodinium oxygen flux, 0.5 ml of the Symbiodinium slurry was mixed with 2.5 ml filtered seawater containing 4 mM NaHCO_3_ and loaded into a water-jacketed glass cell respirometry chamber (StrathKelvin Instruments Ltd). The Symbiodinium samples were stirred with magnetic stir bars and maintained at 29°C with a water recirculator. Oxygen flux measurements were recorded in two chambers simultaneously using Clark-type oxygen electrodes. Respiration was measured in darkness for 10 min before and after a series of irradiance levels. The isolated Symbiodinium were exposed to 13 increasing irradiance levels (0–2200 µmol quanta m^−2^ s^−1^) for 10 minutes per level or until a linear change in oxygen concentration was observed in the chamber. For each sample, the irradiance levels were measured using a 4π quantum sensor (WALZ, Effeltrich, Germany). Oxygen fluxes of isolated Symbiodinium were measured after those of the intact branches, at approximately 15:00 or 16:00 local time.

### Calculation of photosynthetic rates

Net and gross rates of oxygen flux were plotted against irradiance to generate photosynthesis versus irradiance (P-E) graphs for both *in hospite* and isolated Symbiodinium. Coefficients from P-E curves (P_s_, the maximum photosynthetic rate in the absence of photoinhibition; α, the initial slope of the curve; and β the photoinhibition coefficient) were determined for each sample by fitting the equation of Platt et al. [43] using the nlsList function in the nlme package of the R statistical software. Photosynthetic rates were standardized to the surface area of the gorgonian branch, the total number of Symbiodinium, and the total amount of chlorophyll a. α was standardized to the amount of chlorophyll *a*. To calculate the diurnal balance between gross photosynthesis and respiration, we integrated the P-E curve over the irradiance for a 24 h period.

### *Symbiodinium* density

Symbiodinium density in a gorgonian branch was estimated from averaging four replicate hemocytometer counts (0.4 mm^3^ each) of the Symbiodinium cell density aliquot. Oxygen fluxes of gorgonian branches were standardized to the total number of Symbiodinium cells in a branch, which was estimated using the density of cells in the homogenized piece (cells cm^−2^) multiplied by the surface area of the entire branch. For isolated Symbiodinium, the number of cells in the respirometry chamber was estimated using the cell density determined from the Symbiodinium cell density aliquot.

### Chlorophyll content in Symbiodinium

For chlorophyll quantification, the *Symbiodinium* cells in the 1.5 ml aliquot of the *Symbiodinium* slurry were pelleted, and the supernatant was decanted. Chlorophylls were extracted from the *Symbiodinium* cells by adding 950 µl of cold 100% acetone and 50 µl DMSO. After 24 h of extraction at −20°C in the dark, the absorbance of the extract was measured at 630, 660, and 750 nm using an ELYPTICA model ELy-2000 spectrophotometer. Absorbance at 750 nm was subtracted from the absorbance at 630 nm and 660 nm for each sample and chlorophylls a and c_2_ were estimated using the equations of Jeffrey and Humphrey [44]. Chlorophyll concentrations were standardized to surface area (µg chl cm^−2^) and to cell density (pg chl cell^−1^). Oxygen flux of gorgonian branches was standardized to the total amount of chlorophyll *a* in the gorgonian branches, which was calculated using the concentration of chlorophyll per surface area of the homogenized piece multiplied by the surface area of the entire branch. For isolated Symbiodinium, the amount of chlorophyll *a* in the respirometry chamber was estimated using the chlorophyll *a* concentration obtained from the 1.5 ml chlorophyll content aliquot.

### Estimated absorbance and chlorophyll *a* specific absorption

Following the oxygen flux measurements, the reflectance spectrum (400–750 nm) of each branch was measured using an Ocean Optics USB 4000 fiber optic spectrophotometer. The fiberoptic cable was held at a 45° angle above a branch, which was illuminated on all sides to produce a homogeneous light field (designed by T. Scheufen, UNAM). A dried gorgonian branch, painted with white acrylic paint, was used to correct for light scattered by the surface of the gorgonian branch. The painted branch reflected ∼90% of PAR compared to a similarly shaped object wrapped in Teflon. Surface-corrected reflectance spectra were standardized to the reflectance value at 750 nm. Estimated absorbance spectra (D_e_) were calculated as the negative log of the corrected reflectance. Chlorophyll *a* specific absorption (a*_chl *a*_), was calculated using the equation a*_chl *a*_ = (D_e 675_/ρ) x ln(10), where ρ is mg m^−2^ of chlorophyll *a* [45].

### Genetic identification of *Symbiodinium*

For each gorgonian branch, 2 ml of the isolated Symbiodinium slurry was centrifuged at 10,000 rpm for 1 min to pellet the Symbiodinium cells. The supernatant was removed and replaced with 2 ml 100% EtOH to preserve the Symbiodinium. DNA was extracted from an aliquot of this solution using the Qiagen DNeasy Plant Mini kit. The internal transcribed spacer 2 (ITS2) region of ribosomal DNA was amplified using the primers ITSintfor2, ITS-Reverse, and ITS2CLAMP following the protocol of LaJeunesse [34]. The PCR products were separated using denaturing gradient gel electrophoresis (DGGE) with a 45–80% denaturing gradient and run at 120 V for 13 h. Dominant ITS2 sequence variants in unique profiles were excised, re-amplified using ITSintfor2 and ITS-Reverse, and then sequenced using an Applied Biosystems 3730XL automated sequencer at the DNA Laboratory at Arizona State University.

Since we found that at least one colony of each of the four gorgonian species harbored *Symbiodinium* type B1, we determined whether colonies of the four different species harbored the same B1 lineage [36]. Microsatellite flanking regions from B1 *Symbiodinium* were PCR amplified with the primers B7Sym15 [46] and CA4.86 [35] and directly sequenced using an Applied Biosystems 3730XL automated sequencer. The flanking sequences were concatenated and aligned with 16 published *Symbiodinium* type B1 sequences from Supplementary tables S1 in [36], [47]. Six samples, five from our study (1 *P. anceps*, 2 *P. porosa*, 2 *P. wagenaari*) and one published (B1 lineage 1.4) [36], were only represented by B7Sym15 sequences. For each flanking region, the Jukes Cantor model of sequence evolution was chosen using AIC scores from jModelTest (2.1.4) [48], [49] and substitution rates were assumed to follow a gamma distribution with four categories. In order to determine the phylogenetic relationships among B1 *Symbiodinium*, a Bayesian phylogenetic tree was generated using the MrBayes (3.2.2) [50]. Two sets of four independent chains were run for 1,000,000 generations, but the model converged after 916,000 generations. Trees were sampled every 100 generations (916 trees per run) and the first 25% of trees were discarded as burn-in. The average standard deviation of split frequencies was less than 0.01. To confirm the topology generated by MrBayes, 100 bootstrap replicates of a maximum-likelihood phylogenetic tree were generated in Garli 2.0 using the Jukes Cantor model of sequence evolution [51]. *Symbiodinium* type B19, from supplementary table S1 in [36] was used as an outgroup for both analyses.

### Statistical methods

In a given host species, different host-symbiont combinations can differ in their physiology [52]. Therefore, we excluded from statistical analyses gorgonian-*Symbiodinium* ITS2 type combinations represented by three or fewer colonies. The sample sizes given in the results reflect the number of colonies used in the statistical analyses. Each parameter was analyzed using a one-way ANOVA with gorgonian species as a factor. Residuals for most parameters were not normally distributed and/or had unequal variance among species. Therefore, most data required transformation using a log, square root, or reciprocal function. When significant differences were found among gorgonian species, Tukey HSD post hoc tests were used to identify significant differences among all pairwise species combinations using Bonferonni-corrected p-values. a*_chl *a*_ data could not be transformed to meet the assumptions of ANOVA and was tested by determining the frequency of obtaining an F statistic greater than or equal to the observed F statistic in 10,000 permutations of the data.

## Results

### Photochemical efficiency of photosystem II in Symbiodinium

Measuring the effective (Δ*F/F_m_'*) and maximum (*F_v_/F_m_*) quantum yield of PSII throughout the acclimation period enabled us to calculate maximum excitation pressure over photosystem II, *Q_m._ Q_m_* ranged from 0.25 in *P. wagenaari* to 0.32 in *P. anceps* and did not significantly differ between the four species. The *Q_m_* values demonstrated that there was no detrimental tank effect on the gorgonians.

### Oxygen flux in gorgonian branches

**Figure 1:**
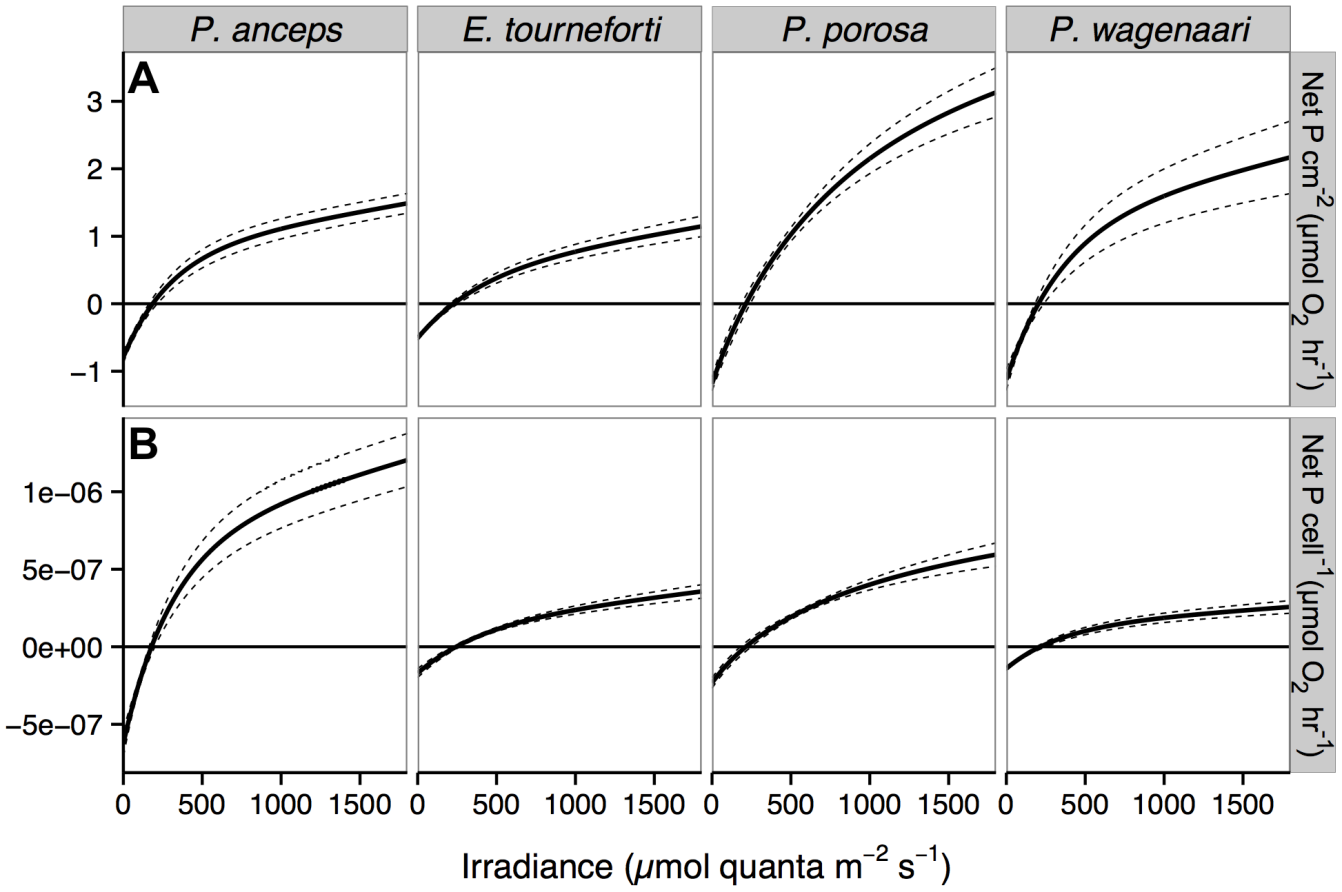
*In hospite* net photosynthesis-irradiance (P-E) curves from four Caribbean gorgonian species. (A) P-E curves per cm^2^ of gorgonian branches and, (B) P-E curves per *Symbiodinium* cell. Solid lines represent the average fitted values for *Pterogorgia anceps* (n = 9), *Eunicea tourneforti* (n = 8), *Pseudoplexaura porosa* (n = 6), and *Pseudoplexaura wagenaari* (n = 6). Dotted lines represent ± standard error. The photosynthetic rate at 1800 µmol quanta was used as a proxy for the maximum photosynthetic rate.

P-E curves for gorgonian branches did not reach saturation despite being exposed to more than 1800 µmol quanta m^−2^ s^−1^. Therefore, the maximum photosynthetic rate could not be determined as in Platt et al. [43]. Since 1800 µmol quanta m^−2^ s^−1^ was the highest irradiance shared between the two respirometric chambers, the fitted values at 1800 µmol quanta m^−2^ s^−1^ were used as a proxy for the maximum photosynthetic rates. P. porosa and P. wagenaari had the highest average photosynthetic rates per cm^2^ at 1800 µmol quanta m^−2^ s^−1^ (Figure 1A), with both species having similar gross photosynthetic and respiration rates per cm^2^, although *P*. porosa had significantly higher net photosynthetic rates per cm^2^ than P. wagenaari (Table 1). *P. anceps* had significantly higher respiration rates per cm^2^ than *E. tourneforti*, but the two species did not differ significantly in their maximum photosynthetic rates per cm^2^ (Figure 1A, Table 1). When integrated on a diurnal cycle, the total oxygen produced via photosynthesis (10.3 to 25.9 µmol O_2_) was less than the oxygen consumed via respiration (13.4 to 29.7 µmol O_2_) for each gorgonian species and therefore the 24 h gross P/R were less than 1 (Table 1).

**Table 1:** Photosynthesis-irradiance (P-E) curve parameters for gorgonian species.

	Gorgonian species				One-way ANOVA			
	*P. anceps* (9)	*E. tourneforti* (8)	*P. porosa* (6)	*P. wagenaari* (6)	MS	F_3, 25_	p	1-β
gross P cm^−2^	2.28^ac^±0.61	1.66^a^±0.48	4.32^b^±1.05	3.30^bc^±1.07	0.22	10.47	<0.001	
net P cm^−2^	1.49^a^±0.44	1.15^a^±0.43	3.13^b^±0.89	2.17^a^±0.70	0.24	8.59	<0.001	
R cm^−2^	−0.79^a^±0.25	−0.51^b^±0.09	−1.19^c^±0.25	−1.12^ac^±0.38	0.19	12.12	<0.001	
gross P µg^−1^ chl *a*	1.04^a^±0.39	0.71^a^±0.18	0.32^b^±0.08	0.15^c^±0.03	0.99	52.60	<0.001	
net P µg^−1^ chl *a*	0.68^a^±0.25	0.48^a^±0.12	0.23^b^±0.05	0.10^c^±0.02	1.00	46.70	<0.001	
gross P cell^−1^ *10^−7^	18.29^a^±7.35	5.27^bc^±1.95	8.28^b^±2.52	3.98^c^±0.70	0.50	27.46	<0.001	
net P cell^−1^ *10^−7^	12.04^a^±5.14	3.57^bc^±1.23	5.95^b^±1.84	2.57^c^±0.40	0.44	22.90	<0.001	
α *10^−3^ (P µg^−1^ chl *a*)	2.51^a^±1.25	1.26^b^±0.57	0.53^c^±0.22	0.32^c^±0.10	1.11	29.36	<0.001	
24 h gross P/R	0.88±0.21	0.78±0.23	0.93±0.16	0.83±0.18	0.03	0.78	0.516	0.22

Sample sizes for each gorgonian species are given in parentheses next to the species name (see Figure 1 legend for full genus names). P and R represent photosynthetic and respiration in µmol O_2_ hr^−1^ rates at 1800 µmol quanta m^−2^ s^−1^, respectively. α represents the initial slope of the P-E curve. Table cells contain the sample mean ± standard deviation. The mean square (MS), F statistics (F), and significance value (p) are from a one-way ANOVA using gorgonian species as a factor. All variables were transformed prior to conducting ANOVA. Means with different superscript letters are statistically different (α = 0.05). Power (1- β) is shown for non-significant results.

The gross and net photosynthetic rates per *Symbiodinium* cell exhibited a different pattern than the photosynthetic rates per cm^2^ (Table 1). *P. anceps* had significantly higher gross and net photosynthetic rates per cell than all other species (Figure 1B, Table 1). *P. porosa* had the second highest average photosynthetic rates per cell, which were significantly higher than those of *P. wagenaari*, but not *E. tourneforti*. Photosynthetic rates per cell of *E. tourneforti* were similar to both *P. porosa* and *P. wagenaari*.

Photosynthetic rates per chlorophyll *a* produced a similar pattern as photosynthetic rates per cell (Table 1). *P. anceps* had the highest average photosynthetic rates per chlorophyll *a* (gross and net), significantly higher than the two *Pseudoplexaura* species, but similar to *E. tourneforti*. *P. wagenaari* had significantly lower rates than *P*. *porosa* (Table 1). Differences in the initial slope of the P-E curve, α, mirrored differences in photosynthetic rates per chlorophyll *a*, as α was significantly greater for *P. anceps* than all other species (Table 1). α of *E. tourneforti* was significantly greater than in both *Pseudoplexaura* species, which had statistically similar α.

### Oxygen flux of isolated *Symbiodinium*

Photosynthetic rates of isolated Symbiodinium were lower than the in hospite photosynthetic rates except for *Symbiodinium* from E. tourneforti (Table 2). With the exception of Symbiodinium from E. tourneforti, isolated Symbiodinium had gross P_max_/R less than or equal to one (Table 2). The initial slopes of the P-E curves (α) were greater for isolated Symbiodinium (Table 2) than for Symbiodinium within gorgonian branches (Table 1).

**Table 2:** Photosynthesis-irradiance (P-E) curve parameters for isolated *Symbiodinium*.

	Gorgonian species				One-way ANOVA		
	*P. anceps* (9)	*E. tourneforti* (8)	*P. porosa* (6)	*P. wagenaari* (6)	MS	F_3, 25_	p
net P_max_ µg^−1^ chl *a*	−0.01^a^±0.06	0.36^b^±0.21	−0.01^a^±0.01	0.01^a^±0.02	0.03	27.47	<0.001
net P_max_ cell^−1^ *10^7^	−0.00^a^±1.10	2.47^b^±1.47	−0.32^a^±0.18	0.08^a^±0.36	0.30	7.66	<0.001
R cell^−1^ *10^7^	−2.27^a^±0.96	−1.81^ac^±0.99	−0.62^b^±0.28	−1.06^bc^±0.48	0.12	10.01	<0.001
α *10^−3^ (P µg^−1^ chl *a*)	4.23^ab^±2.37	7.57^a^±4.71	2.01^bc^±1.25	1.10^c^±0.62	0.45	13.19	<0.001
gross P_max_/R	0.41^a^±0.18	2.65^b^±0.55	0.15^a^±0.09	0.83^a^±0.46	0.16	16.30	<0.001

Sample sizes for *Symbiodinium* isolates from each gorgonian species (see Figure 1 legend for full genus names) are given in parentheses next to the species' name. P_max_ and R represent the maximum photosynthetic and respiration rates, respectively, in µmol O_2_ hr^−1^. α is the initial slope of the P-E curve. Table cells contain the sample mean ± standard deviation. The mean square (MS), F statistics (F), and significance value (p) from a one-way ANOVA using gorgonian species as a factor are shown for each variable. All variables were transformed prior to conducting the ANOVA. Means with different superscript letters are statistically different (α = 0.05).

### *Symbiodinium* density

**Figure 2:**
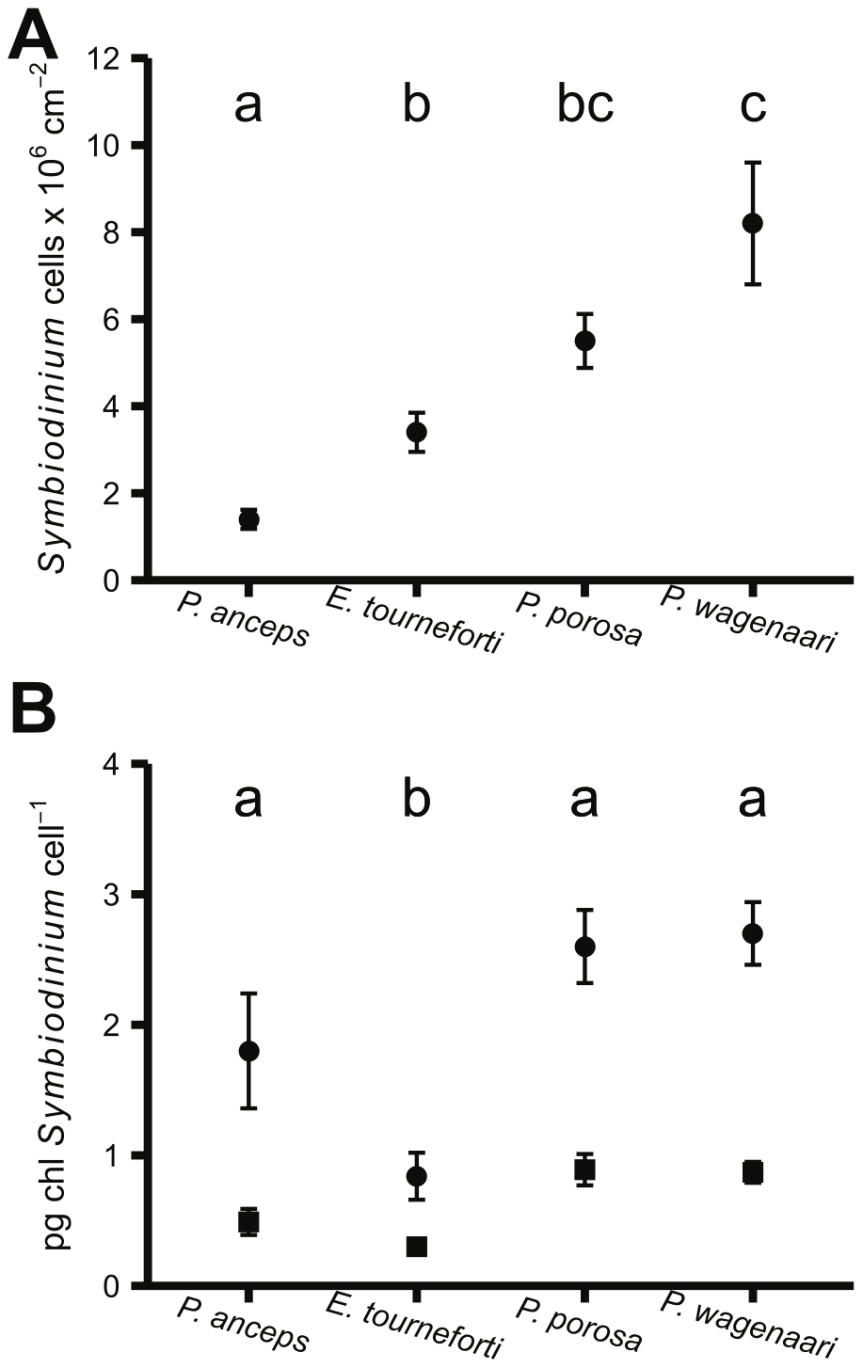
*Symbiodinium* parameters in four Caribbean gorgonian species. (A) Cell densities and, (B) Concentration of chlorophylls *a* (circles) and *c*_2_ (squares) per *Symbiodinium* cell. Points represent sample means ± standard error. Gorgonian species that do not share a letter are significantly different from each other in either density or chlorophyll *a* per cell (α = 0.05, see Figure 1 for full species names and sample sizes). See Table 3 for significant differences in chlorophyll *c*_2_ per cell.

P. wagenaari had the highest Symbiodinium densities and these differed significantly from the Symbiodinium densities in *P. anceps* and *E. tourneforti* (Figure 2A, Table 3). *P. anceps* had the lowest *Symbiodinium* densities.

**Table 3:** Photosynthetic characteristics of *Symbiodinium* in four Caribbean gorgonian species.

	Gorgonian species				One-way ANOVA			
	*P. anceps* (9)	*E. tourneforti* (8)	*P. porosa* (6)	*P. wagenaari* (6)	MS	F_3, 25_	p	1-β
*Sym* ITS2 type	B1	B1L	B1i	B1				
*Sym* density (10^6^ cells cm^−2^)	1.43^a^±0.67	3.45^b^±1.3	5.47^bc^±1.53	8.20^c^±3.43	0.84	26.62	<0.001	
chl *a* (pg cell^−1^)	1.80^a^±1.32	0.84^b^±0.51	2.62^a^±0.69	2.70^a^±0.60	0.49	10.40	<0.001	
chl *c*_2_ (pg cell^−1^)	0.49^ab^±0.31	0.31^a^±0.16	0.89^c^±0.30	0.87^bc^±0.19	0.38	10.90	<0.001	
chl *a*/chl *c*_2_	3.56^a^±0.40	2.65^b^±0.67	2.99^ab^±0.30	3.10^ab^±0.25	1.19	5.83	0.004	
chl *a* (µg cm^−2^)	2.13^a^±1.01	2.55^a^±1.33	13.85^b^±3.81	21.38^b^±4.60	1.80	75.38	<0.001	
chl *c*_2_ (µg cm^−2^)	0.59^a^±0.26	1.00^a^±0.56	4.68^b^±1.41	6.92^c^±1.53	63.49	67.60	<0.001	
D_e 675_	0.49^a^±0.05	0.40^a^±0.08	0.65^b^±0.14	0.51^ab^±0.08	0.07	9.23	<0.001	
*Q*_m_	0.32±0.18	0.28±0.06	0.29±0.07	0.25±0.11	0.01	0.41	0.745	0.133

Sample sizes for each gorgonian species (see Figure 1 legend for full genus names) are given in parentheses next to the species name. Parameters include the abundant *Symbiodinium* (*Sym*) internal transcribed spacer region two (*Sym* ITS2) type, *Sym* cell density, chlorophyll content (chl), estimated absorbance at 675 nm (D_e 675_), as well as the pressure over photosystem II (*Q_m_*). Table cells contain the sample mean ± standard deviation. The mean square (MS), F statistics (F), and significance value (p) from a one-way ANOVA, using gorgonian species as a factor, are shown for each variable. All variables were transformed prior to conducting the ANOVA. Means with different superscript letters were statistically different (α = 0.05) and this was diagnosed by Tukey's HSD post hoc tests. Power (1- β) is shown for non-significant results.

### Chlorophyll content

*P.* porosa and P. wagenaari had significantly more chlorophyll a and *c*_2_ per cm^2^ than P. anceps and E. tourneforti (Table 3). On the other hand, P. porosa, P. wagenaari, and P. anceps had statistically similar chlorophyll a per Symbiodinium cell, with Symbiodinium in E. tourneforti having significantly lower chlorophyll a per cell than all other Symbiodinium (Figure 2B, Table 3). While chlorophyll *c*_2_ per Symbiodinium cell exhibited a similar pattern to chlorophyll *a* per cell, the statistical results were slightly different: Symbiodinium in *P. porosa* had significantly greater chlorophyll *c*_2_ per cell than those in *P. anceps* (Figure 2B, Table 3). *Symbiodinium* in *E. tourneforti* had lower chlorophyll *c*_2_ per cell than *Symbiodinium* in both *Pseudoplexuara* species, but similar chlorophyll *c*_2_ per cell as those in *P. anceps* (Figure 2B, Table 3). *Symbiodinium* in P. porosa and P. wagenaari had statistically similar ratio of chlorophyll a to chlorophyll c_2_ to those in *P. anceps* and *E. tourneforti*, but *Symbiodinium* in *E. tourneforti* had a significantly lower ratio of chlorophyll a to chlorophyll c_2_ than those in P. anceps (Table 3).

### Chlorophyll *a* specific absorption

**Figure 3:**
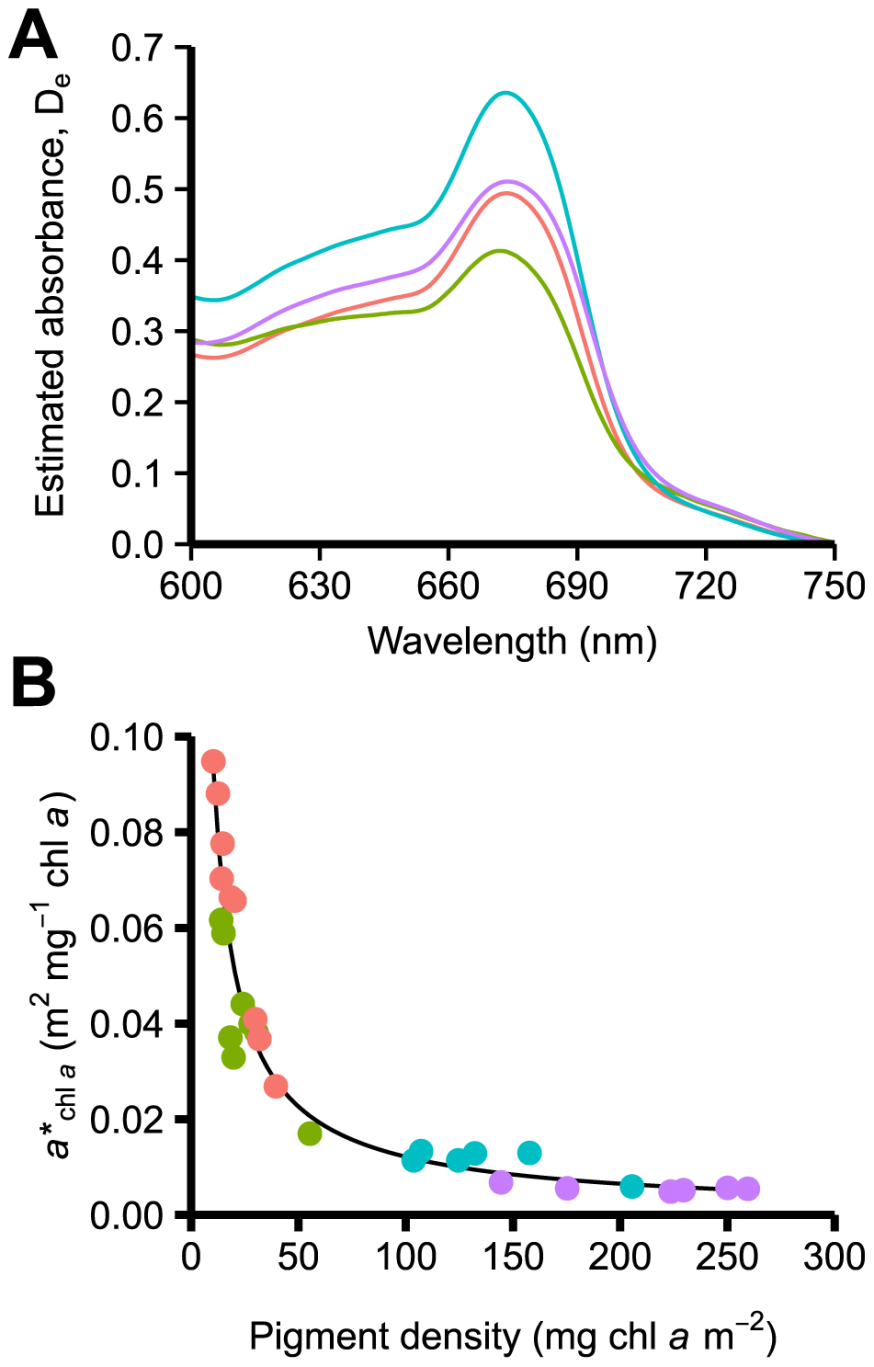
Estimated absorbance spectra, D_e_ (A), and Chlorophyll *a* specific absorption, a*_chl *a*_ (B) in four Caribbean gorgonian species. *Pterogorgia anceps* (red line; n = 9), *Eunicea tourneforti* (green line; n = 8), *Pseudoplexaura porosa* (blue line; n = 6), and *Pseudoplexaura wagenaari* (purple line; n = 6). Lines in (A) represent average D_e_ spectra for each species. The equation for the line in (B) is y = 0.7586*(x^−0.8976^).

P. porosa had the highest estimated absorbance at 675 nm (D_c 675_), which was significantly greater than that in *P. anceps* and E. tourneforti, but similar to *P. wagenaari* (Figure 3A, Table 3). Consistent with chlorophyll density data, chlorophyll *a* specific absorption (a*_chl *a*_), was significantly higher in P. anceps and E. tourneforti than in the two Pseudoplexaura species (Figure 3B, Table 3). P. wagenaari had significantly lower a*_chl *a*_ than all other species.

### Genetic identification of *Symbiodinium*

All P. anceps colonies harbored B1 Symbiodinium, matching accession AF333511 [32]. Eight of the nine E. tourneforti colonies sampled harbored type B1L, matching accession GU907639 [36], while one colony harbored type B1 matching accession AF333511 [32]. Six P. porosa colonies harbored Symbiodinium B1i matching accession GU907636 [36]. The remaining three P. porosa colonies harbored one of two distinct DGGE profiles with dominant ITS2 sequences identical to type B1 [32]. All P. wagenaari colonies harbored type B1 Symbiodinium, albeit with a distinct DGGE profile from the B1 symbiont in P. anceps (Figure S1) as discussed in [36].

**Figure 4:**
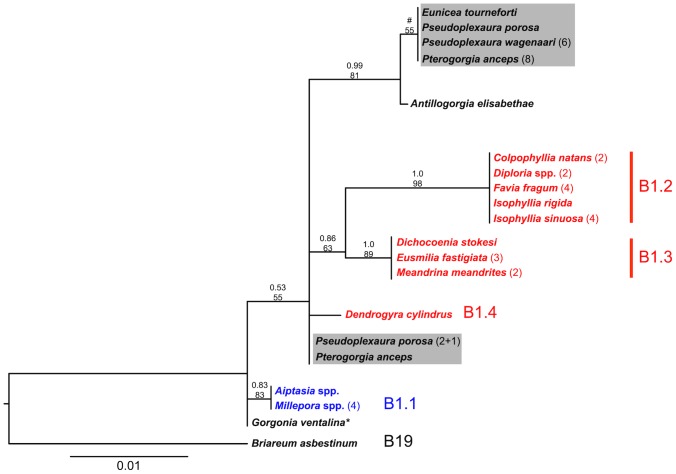
A maximum likelihood phylogenetic tree based on microsatellite flanking regions of B1 *Symbiodinium*. The phylogeny includes B1 *Symbiodinium* from the four gorgonian species in this study (highlighted in gray), from other gorgonian corals [47], from scleractinian and hydrozoan corals [36], as well as *Symbiodinium minutum* from *Aiptasia*, a sea anemone [47]. Branch tips are labeled with host species and sample sizes when n>1. Gorgonian and scleractinian coral species are shown in black and red, respectively, and the other cnidarians are shown in blue. B1 lineages described by Finney et al. [36] are listed besides the host taxa. Numbers above the branches are the posterior probability above the maximum likelihood consensus support for each group. B1 *Symbiodinium* from 16 of 19 gorgonian colonies sampled clustered in a phylogenetic group with high posterior probability (top gray box). Three gorgonian colonies were placed outside of this clade (bottom gray box) and were most closely related to *Symbiodinium* isolated from *Pseudoplexaura porosa* from Florida (indicated with (+1)) and cultured *Symbiodinium* from *Gorgonia ventalina* [47] indicated with *. (#) indicates a group recovered in the maximum likelihood tree, but not the Bayesian phylogenetic tree.

Since at least one colony from all the four studied gorgonian species hosted *Symbiodinium* type B1, we compared these B1 *Symbiodinium* amongst the four gorgonian species in our study and to published B1 sequences from other cnidarians. Analysis of microsatellite flanking region sequences revealed that *Symbiodinium* B1 from 16 of 19 gorgonian colonies, representing all four gorgonian species, formed a phylogenetic group with high posterior probability (Figure 4). The B1 *Symbiodinium* in this group included *Symbiodinium* from eight of nine *P. anceps* colonies, one colony each of *E. tourneforti* and *P. porosa* and all six *P. wagenaari* colonies. B1 *Symbiodinium* from three gorgonian colonies (from *P. anceps* and *P. porosa*) placed outside this group, but did not cluster with the examined *Symbiodinium* B1 lineages from scleractinian corals.

## Discussion

Gorgonian corals blanket the landscape of Caribbean coral reefs [1], [2], [7], yet few data exist on the physiology of their mutualism with *Symbiodinium* [24]–[29]. Unlike scleractinian corals, whose abundance has dramatically declined in the Caribbean [7]–[11], gorgonians have withstood recent environmental perturbations. Learning about gorgonian symbioses, at current ambient conditions, enhances our understanding and our ability to predict the future of Caribbean coral reefs. We therefore employed multiple tools to characterize aspects of *Symbiodinium* photosynthesis in four common Caribbean gorgonian species.

Oxygen flux data demonstrated differences in the *Symbiodinium* in the four studied gorgonians. For example, the two *Pseudoplexaura* species had the highest average photosynthetic rates per cm^2^, probably due to the higher *Symbiodinium* and chlorophyll densities compared to *P. anceps* and *E. tourneforti*. On the other hand, the two *Pseudoplexaura* species had lower initial slopes of the P-E curves, low photosynthetic rates per *Symbiodinium* cell and per chlorophyll *a*, and lower chlorophyll *a* specific absorption. Taken together, these data suggest that *Symbiodinium* in *P. porosa* and *P. wagenaari* are less efficient in light absorbtion and utilization than *Symbiodinium* in *P. anceps* and *E. tourneforti*. The light levels available for *Symbiodinium* could differ due to *Symbiodinium* self-shading or to host tissue characteristics such as tissue thickness [53], [54] or pigmentation.

The possibility that *Symbiodinium* in *P. porosa* and *P. wagenaari* are less efficient in light absorbtion and utilization is corroborated by looking at changes in chlorophyll a specific absorption coefficient as a function of chlorophyll *a* density. In the two *Pseudoplexaura* species, the a*_chl *a*_ values as a function of chlorophyll *a* density were very low, comparable to those values reported for phytoplankton and freshly isolated *Symbiodinium* from *Porites banneri* [45], [55]. On the other hand, E. tourneforti a*_chl *a*_ values fell within those previously reported for scleractinian corals. Lastly, the a*_chl *a*_ values of *Symbiodinium* in *P. anceps* demonstrated a very high pigment light absorption efficiency, comparable to that of *Symbiodinium* in the scleractinian coral *Porites banneri* [45].

Of the four gorgonian species, *Symbiodinium* in *P. anceps* exhibited twice the photosynthetic rate per *Symbiodinium* cell than that of the next gorgonian species (*P. porosa*) and the highest average photosynthetic rates per chlorophyll *a*. The relatively high photosynthesis per *Symbiodinium* in *P. anceps* may be related to the low density of *Symbiodinium* and chlorophyll *a* per cm^2^. In addition, the thin, angular branches and low polyp density may aid *Symbiodinium* photosynthesis in *P. anceps* by maximizing gas exchange and/or reducing self-shading of *Symbiodinium*.

The four Caribbean gorgonian species produced comparable photosynthetic and respiration rates per cm^2^ to the average rates of eight shallow scleractinian coral species (P = 2.0, R = 0.64) [39]. Conversely, the Mediterranean gorgonian *Eunicella singularis* at 15 m depth had lower average *Symbiodinium* photosynthetic (∼1) and respiration (∼0.55) rates per cm^2^ [56]. The differences could be due to *E. singularis* in deeper waters being exposed to lower irradiance levels than those in the current study [56], [57]. In our study, the four gorgonian species did not exhibit photoinhibition, similar to what has been observed in other symbioses between *Symbiodinium* and cnidarians [58]. The lack of photoinhibition may be due to branch tissue thickness, as was seen in the octocoral *Capnella gaboensis* [53], [59]. The gross P/R ratios in the four gorgonian symbioses, ranging from 2 to 4, were comparable to ratios for other octocorals [60]–[63], anemones [52], and shallow water scleractinian corals [39]. On the other hand, the ratios of diurnal integrated gross photosynthesis to respiration were below 1. It remains to be determined the extent of the contribution of the *Symbiodinium* autotrophic production to the energy budget of these Caribbean gorgonians.

*Symbiodinium* photosynthesis within a host may also be affected by host characteristics [54], [64]. For example, the scleractinian coral skeleton enhances light absorption by *Symbiodinium* [45], [65], and *Symbiodinium* chlorophyll *a* specific absorption is higher in symbiosis than in isolation [45]. Chlorophyll *a* specific absorption of *Symbiodinium* in the studied gorgonian corals was comparable to that in scleractinian corals [45], [54], even though gorgonian skeletal structure (sclerites and an axial rod) substantially differs from the calyx structure in scleractinian corals. The calcite sclerites within gorgonian tissues may produce the same effect as the light scattering of the scleractinian coral skeleton, perhaps similar to the influence of the siliceous spicules of sponges on light transmission [66], [67].

Furthermore, in symbioses between cnidarians and Symbiodinium, photosynthesis is dependent upon the genetic identities of both the host and symbiont [52]. Therefore, it is imperative to identify the Symbiodinium. In the Caribbean, scleractinian corals host Symbiodinium belonging to clades A, B, C, or D [34], [36]. On the other hand, the majority of Caribbean gorgonian species host only clade B Symbiodinium [33], [68]. The four gorgonian species in this study were no exception; harboring clade B Symbiodinium belonging to types B1, B1i, and B1L. Types B1i and B1L have only been reported in Caribbean gorgonian species [36], [69]. Sequencing of the B1 Symbiodinium from the four gorgonian species revealed that they harbor B1 Symbiodinium with distinct microsatellite flanking region sequences from those in scleractinian coral species. The B1 *Symbiodinium* obtained from most gorgonian colonies in our study formed a distinct group amongst the previously identified B1 lineages from scleractinian corals (Figure 4).

Although both *P. anceps* and *P*. *wagenaari* hosted type B1 Symbiodinium, the photosynthetic characteristics differed between these two symbioses. Photosynthetic variability within *Symbiodinium* type B1 has also been observed in cultures [70], and may be associated with distinct genetic lineages within type B1 [35], [36]. In our study, eight of the nine *P. anceps* colonies, and all *P. wagenaari* colonies, harbored symbionts from the same, highly supported, phylogenetic group within B1 *Symbiodinium*. Therefore, the observed photosynthetic variability between *P. anceps* and *P*. *wagenaari* was not due to different B1 lineages but probably due to the physiology of different host-symbiont combinations [52].

In *E. tourneforti*, Symbiodinium had comparable photosynthetic rates per cell in the intact symbiosis and in isolation. On the other hand, maximum photosynthetic rates per cell in *Symbiodinium* isolated from *P. anceps, P. porosa*, and *P. wagenaari* were lower than those measured *in hospite*, although the average α was higher in isolation. Diminished photosynthetic rates in isolated Symbiodinium cells may occur in the absence of host carbon concentrating mechanisms [71] or differences in carbonic anhydrase activity among *Symbiodinium* types [72]. Exposure to the ionic environment of seawater [73], [74] or bacteria [75] may also reduce photosynthesis in isolated Symbiodinium. In the sea anemone *Aiptasia pallida*, at ambient temperatures, the photosynthetic rates of isolated Symbiodinium were also lower compared to the intact association [52], although not to the degree measured here. Conversely, secondary metabolites released from homogenized gorgonian corals may impair isolated Symbiodinium, as reported for the homogenate of the soft coral *C. gaboensis* that lysed Symbiodinium cells [76]. Therefore, secondary metabolites produced by many gorgonians may limit the utility of investigating freshly isolated Symbiodinium.

In conclusion, our results contribute to consequential data on Symbiodinium physiological performance in their mutualism with four Caribbean gorgonian species at ambient temperature. Given that gorgonian corals are either maintaining or increasing their abundance on Caribbean coral reefs, understanding aspects of their symbiosis is imperative to understanding the future of Caribbean coral reefs. This study demonstrates differences between Symbiodinium photosynthetic characteristics in the four gorgonian species, collected from the same site, maintained under identical conditions, and with two of the gorgonian species containing the same *Symbiodinium* type. The differences observed between the gorgonian symbioses emphasize the influence of the host physiology and architecture on *Symbiodinium* photosynthesis.

## Supporting Information

Figure S1 **Denaturing gradient gel electrophoresis gel of ITS2 DNA from** ***Symbiodinium*** **associated with** ***Pseudoplexaura porosa*** **,** ***Pseudoplexaura wagenaari*** **, and** ***Pterogorgia anceps*** **.** Type B1 *Symbiodinium* was recovered from all colonies of *P. wagenaari* and *P. anceps*, but from only 3 of 9 *P. porosa* colonies. B1 *Symbiodinium* exhibited distinct DGGE profiles. ($) denotes a band that is faint in these samples, but is typically visible in B1 *Symbiodinium* from *P. anceps*. (*) denotes the band of the type B1i ITS2 sequence variant.
(PDF)

## References

[1] Bayer FM (1961) The shallow-water Octocorallia of the West Indian region. Studies on the Fauna of Curaçao and other Caribbean Islands 12: 1–428.

[2] Cary LR (1918) The Gorgonaceae as a factor in the formation of coral reefs: Carnegie Institution of Washington.

[3] Kinzie I, Robert A (1973) The zonation of West Indian gorgonians. Bull Mar Sci 23: 93–155.

[4] Lasker HR (1985) Prey preferences and browsing pressure of the butterflyfish *Chaetodon capistratus* on Caribbean gorgonians. Mar Ecol Prog Ser 21: 213–220.

[5] Lasker HR, Coffroth MA, Fitzgerald LM (1988) Foraging patterns of *Cyphoma gibbosum* on octocorals: the roles of host choice and feeding preference. Biol Bull 174: 254–266.

[6] Vreeland HV, Lasker HR (1989) Selective feeding of the polychaete *Hermodice carunculata* Pallas on Caribbean gorgonians. J Exp Mar Bio Ecol 129: 265–277.

[7] Ruzicka RR, Colella MA, Porter JW, Morrison JM, Kidney JA, et al (2013) Temporal changes in benthic assemblages on Florida Keys reefs 11 years after the 1997/1998 El Niño. Mar Ecol Prog Ser 489: 125–141.

[8] Hughes TP (1994) Catastrophes, phase shifts, and large-scale degradation of a Caribbean coral reef. Science 265: 1547–1551. 17801530 10.1126/science.265.5178.1547

[9] Gardner TA, Côte IM, Gill JA, Grant A, Watkinson AR (2003) Long-term region-wide declines in Caribbean corals. Science 301: 958–960. 12869698 10.1126/science.1086050

[10] Pandolfi JM, Jackson JBC, Baron N, Bradbury RH, Guzman HM, et al (2005) Are U.S. coral reefs on the slippery slope to slime? Science 307: 1725–1726. 15774744 10.1126/science.1104258

[11] Edmunds PJ, Elahi R (2007) The demographics of a 15-year decline in cover of the Caribbean reef coral *Montastraea annularis* . Ecol Monogr 77: 3–18.

[12] Miller SL, Chiappone M, Rutten LM (2009) Large-scale assessment of the abundance, distribution, and condition of benthic coral reef organisms in the Florida Keys National Marine Sanctuary - 2009 Quick look report and data summary: CMS/UNCW, Key Largo FL. 329 p.

[13] Colvard NB, Edmunds PJ (2011) Decadal-scale changes in abundance of non-scleractinian invertebrates on a Caribbean coral reef. J Exp Mar Bio Ecol 397: 153–160.

[14] Dahlgren EJ (1989) Gorgonian community structure and reef zonation patterns on Yucatan coral reefs. Bull Mar Sci 45: 678–696.

[15] Murdock GR (1978) Circulation and digestion of food in the gastrovascular system of gorgonian octocorals (Cnidaria; Anthozoa). Bull Mar Sci 28: 363–370.

[16] Murdock GR (1978) Digestion, assimilation, and transport of food in the gastrovascular cavity of a gorgonian octocoral (Cnidaria; Anthozoa). Bull Mar Sci 28: 354–362.

[17] Goldberg WM, Benayahu Y (1987) Spicule formation in the gorgonian coral *Pseudoplexaura flagellosa*. 1: Demonstration of intracellular and extracellular growth and the effect of ruthenium red during decalcification. Bull Mar Sci 40: 287–303.

[18] Goldberg WM, Benayahu Y (1987) Spicule formation in the gorgonian coral *Pseudoplexaura flagellosa*. 2: Calcium localization by antimonate precipitation. Bull Mar Sci 40: 304–310.

[19] Brazeau DA, Lasker HR (1992) Growth rates and growth strategy in a clonal marine invertebrate, the Caribbean octocoral *Briareum asbestinum* . Biol Bull 183: 269–277. 29300669 10.2307/1542214

[20] Lasker HR, Boller ML, Castanaro J, Sánchez JA (2003) Determinate growth and modularity in a gorgonian octocoral. Biol Bull 205: 319–330. 14672986 10.2307/1543295

[21] Lasker HR (1981) A comparison of the particulate feeding abilities of three species of gorgonian soft coral. Mar Ecol Prog Ser 5: 61–67.

[22] Ribes M, Coma R, Gili J-M (1998) Heterotrophic feeding by gorgonian corals with symbiotic zooxanthella. Limnol Oceanogr 43: 1170–1179.

[23] Rodríguez AD (1995) The natural products chemistry of West Indian gorgonian octocorals. Tetrahedron 51: 4751–4618. 10.1016/0040-4020(95)00216-U PMC7131365 32287414

[24] Burkholder PR, Burkholder LM (1960) Photosynthesis in some Alcyonacean corals. Am J Bot 47: 866–872.

[25] Kanwisher JW, Wainwright SA (1967) Oxygen balance in some reef corals. Biol Bull 133: 378–390.

[26] Lewis JB, Post EE (1982) Respiration and energetics in West Indian Gorgonacea (Anthozoa, Octocorallia). Comp Biochem Physiol A Comp Physiol 71: 457–459.

[27] Drohan AF, Thoney DA, Baker AC (2005) Synergistic effect of high temperature and ultraviolet-B radiation on the gorgonian *Eunicea tourneforti* (Octocorallia: Alcyonacea: Plexauridae). Bull Mar Sci 77: 257–266.

[28] Mydlarz LD, Jacobs RS (2006) An inducible release of reactive oxygen radicals in four species of gorgonian corals. Mar Freshw Behav Phy 39: 143–152.

[29] Baker DM, Kim K, Andras JP, Sparks JP (2011) Light-mediated ^15^N fractionation in Caribbean gorgonian octocorals: implications for pollution monitoring. Coral Reefs 30: 709–717.

[30] Pochon X, Gates RD (2010) A new *Symbiodinium* clade (Dinophyceae) from soritid foraminifera in Hawai'i. Mol Phylogen Evol 56: 492–497. 10.1016/j.ympev.2010.03.040 20371383

[31] Jeong HJ, Lee SY, Kang NS, Yoo YD, Lim AS, et al (2014) Genetics and morphology characterize the dinoflagellate *Symbiodinium voratum*, n. sp., (Dinophyceae) as the sole representative of *Symbiodinium* clade E. J Eukaryot Microbiol 61: 75–94. 10.1111/jeu.12088 24460699

[32] LaJeunesse TC (2001) Investigating the biodiversity, ecology, and phylogeny of endosymbiotic dinoflagellates in the genus *Symbiodinium* using the ITS region: in search of a "species" level marker. J Phycol 37: 866–880.

[33] Goulet TL, Simmons C, Goulet D (2008) Worldwide biogeography of *Symbiodinium* in tropical octocorals. Mar Ecol Prog Ser 355: 45–58.

[34] LaJeunesse TC (2002) Diversity and community structure of symbiotic dinoflagellates from Caribbean coral reefs. Mar Biol 141: 387–400.

[35] Santos SR, Shearer TL, Hannes AR, Coffroth MA (2004) Fine-scale diversity and specificity in the most prevalent lineage of symbiotic dinoflagellates (*Symbiodinium*, Dinophyceae) of the Caribbean. Molec Ecol 13: 459–469. 14717900 10.1046/j.1365-294x.2003.02058.x

[36] Finney JC, Pettay DT, Sampayo EM, Warner ME, Oxenford HA, et al (2010) The relative significance of host–habitat, depth, and geography on the ecology, endemism, and speciation of coral endosymbionts in the genus *Symbiodinium* . Microb Ecol 60: 250–263. 20502891 10.1007/s00248-010-9681-y

[37] Sampayo EM, Franceschinis L, Hoegh-Guldberg O, Dove SG (2007) Niche partitioning of closely related symbiotic dinoflagellates. Mol Ecol 16: 3721–3733. 17845444 10.1111/j.1365-294X.2007.03403.x

[38] Sampayo EM, Ridgway T, Bongaerts P, Hoegh-Guldberg O (2008) Bleaching susceptibility and mortality of corals are determined by fine-scale differences in symbiont type. Proc Natl Acad Sci U S A 105: 10444–10449. 18645181 10.1073/pnas.0708049105 PMC2492480

[39] Edmunds PJ, Putnam HM, Nisbet RM, Muller EB (2011) Benchmarks in organism performance and their use in comparative analyses. Oecologia 167: 379–390. 21553265 10.1007/s00442-011-2004-2

[40] Imbs A, Latyshev N, Dautova T, Latypov Y (2010) Distribution of lipids and fatty acids in corals by their taxonomic position and presence of zooxanthellae. Mar Ecol Prog Ser 409: 65–75.

[41] Iglesias-Prieto R, Beltran VH, LaJeunesse TC, Reyes-Bonilla H, Thome PE (2004) Different algal symbionts explain the vertical distribution of dominant reef corals in the eastern Pacific. Proc R Soc Lond 271: 1757–1763. 10.1098/rspb.2004.2757 PMC1691786 15306298

[42] Iglesias-Prieto R, Trench RK (1994) Acclimation and adaptation to irradiance in symbiotic dinoflagellates. 1. Responses of the photosynthetic unit to changes in photon flux density. Mar Ecol Prog Ser 113: 163–175.

[43] Platt T, Gallegos CL, Harrison WG (1980) Photoinhibition of photosynthesis in natural assemblages of marine phytoplankton. J Mar Res 38: 687–701.

[44] Jeffrey SW, Humphrey GF (1975) New spectrophotometric equations for determining chlorophylls *a*, *b*, *c* _1_ and *c* _2_ in higher plants, algae and natural phytoplankton. Biochemie und Physiologie der Pflanzen (BPP) 167: 191–194.

[45] Enríquez S, Méndez ER, Iglesias-Prieto R (2005) Multiple scattering on coral skeletons enhances light absorption by symbiotic algae. Limnol Oceanogr: 1025–1032.

[46] Pettay DT, LaJeunesse TC (2007) Microsatellites from clade B *Symbiodinium* spp. specialized for Caribbean corals in the genus *Madracis* . Mol Ecol Notes 7: 1271–1274.

[47] Thornhill DJ, Xiang Y, Pettay DT, Zhong M, Santos SR (2013) Population genetic data of a model symbiotic cnidarian system reveal remarkable symbiotic specificity and vectored introductions across ocean basins. Molec Ecol 22: 4499–4515. 23980764 10.1111/mec.12416

[48] Posada D (2008) jModelTest: phylogenetic model averaging. Mol Biol Evol 25: 1253–1256. 18397919 10.1093/molbev/msn083

[49] Posada D (2009) Selection of models of DNA evolution with jModelTest. Methods in molecular biology (Clifton, NJ) 537: 93–112. 10.1007/978-1-59745-251-9_5 19378141

[50] Huelsenbeck JP, Ronquist F (2001) MRBAYES: Bayesian inference of phylogenetic trees. Bioinformatics (Oxford, England) 17: 754–755. 10.1093/bioinformatics/17.8.754 11524383

[51] Zwickl DJ (2006) Genetic algorithm approaches for the phylogenetic analysis of large biological sequence datasets under the maximum likelihood criterion: The University of Texas at Austin.

[52] Goulet TL, Cook CB, Goulet D (2005) Effect of short-term exposure to elevated temperatures and light levels on photosynthesis of different host-symbiont combinations in the *Aiptasia pallida*/*Symbiodinium* symbiosis. Limnol Oceanogr 50: 1490–1498.

[53] Ramus J, Beale SI, Mauzerall D (1976) Correlation of changes in pigment content with photosynthetic capacity of seaweeds as a function of water depth. Mar Biol 37: 231–238.

[54] Kaniewska P, Magnusson SH, Anthony KRN, Reef R, Kühl M, et al (2011) Importance of macro- versus microstructure in modulating light levels inside coral colonies. J Phycol 47: 846–860. 27020021 10.1111/j.1529-8817.2011.01021.x

[55] Bricaud A, Babin M, Morel A, Claustre H (1995) Variability in the chlorophyll-specific absorption coefficients of natural phytoplankton: Analysis and parameterization. J Geophys Res Oceans 100: 13321–13332.

[56] Ezzat L, Merle P-L, Furla P, Buttler A, Ferrier-Pages C (2013) The response of the Mediterranean gorgonian *Eunicella singularis* to thermal stress is independent of its nutritional regime. PLoS ONE 8: e64370. 23667711 10.1371/journal.pone.0064370 PMC3648542

[57] Ferrier-Pages C, Tambutté E, Zamoum T, Segonds N, Merle P-L, et al (2009) Physiological response of the symbiotic gorgonian *Eunicella singularis* to a long-term temperature increase. J Exp Biol 212: 3007–3015. 19717684 10.1242/jeb.031823

[58] Fitt WK, Cook CB (2001) Photoacclimation and the effect of the symbiotic environment on the photosynthetic response of symbiotic dinoflagellates in the tropical marine hydroid *Myrionema amboinense* . J Exp Mar Bio Ecol 256: 15–31. 11137502 10.1016/s0022-0981(00)00302-6

[59] Farrant PA, Borowitzka MA, Hinde R, King RJ (1987) Nutrition of the temperate Australian soft coral *Capnella gaboensis* 1. Photosynthesis and carbon fixation. Mar Biol 95: 565–574.

[60] Mergner H, Svoboda A (1977) Productivity and seasonal changes in selected reef areas in the Gulf of Aqaba (Red Sea). Helgoländer wiss Meeresunters 30: 383–399.

[61] Fabricius KE, Klumpp DW (1995) Widespread mixotrophy in reef-inhabiting soft corals: the influence of depth, and colony expansion and contraction on photosynthesis. Mar Ecol Prog Ser 125: 195–204.

[62] Tunn KPP, Chou LM, Cheshire AC (1996) Photophysiological studies of the soft coral *Sinularia* in the turbid waters of Singapore. In: Morton B, editor. The Marine Biology of the South China Sea III: Proceedings of the Third International Conference on the Marine Biology of the South China Sea. Hong Kong: Hong Kong University Press. pp. 275–275.

[63] Khalesi MK, Beeftink HH, Wijffels RH (2011) Energy budget for the cultured, zooxanthellate octocoral *Sinularia flexibilis* . Mar Biotechnol 13: 1092–1098. 21537947 10.1007/s10126-011-9373-8

[64] Kaniewska P, Anthony KRN, Hoegh-Guldberg O (2008) Variation in colony geometry modulates internal light levels in branching corals, *Acropora humilis* and *Stylophora pistillata* . Mar Biol 155: 649–660.

[65] Kühl M, Cohen Y, Dalsgaard T, Jørgensen BB, Revsbech NP (1995) Microenvironment and photosynthesis of zooxanthellae in scleractinian corals studied with microsensors for O_2_, pH and light. Mar Ecol Prog Ser 117: 159–172.

[66] Aizenberg J, Sundar VC, Yablon AD, Weaver JC, Chen G (2004) Biological glass fibers: correlation between optical and structural properties. Proc Natl Acad Sci USA 101: 3358–3363. 14993612 10.1073/pnas.0307843101 PMC373466

[67] Brümmer F, Pfannkuchen M, Baltz A, Hauser T, Thiel V (2008) Light inside sponges. J Exp Mar Biol Ecol 367: 61–64.

[68] Goulet TL, Coffroth MA (2004) The genetic identity of dinoflagellate symbionts in Caribbean octocorals. Coral Reefs 23: 465–472.

[69] Santos SR, Taylor DJ, Coffroth MA (2001) Genetic comparisons of freshly isolated vs. cultured symbiotic dinoflagellates: implications for extrapolating to the intact symbiosis. J Phycol 37: 900–912.

[70] Hennige SJ, Suggett DJ, Warner ME, McDougall KE, Smith DJ (2009) Photobiology of *Symbiodinium* revisited: bio-physical and bio-optical signatures. Coral Reefs 28: 179–195.

[71] Goiran C, Al-Moghrabi SM, Allemand D, Jaubert J (1996) Inorganic carbon uptake for photosynthesis by the symbiotic coral/dinoflagellate association I. Photosynthetic performances of symbionts and dependence on sea water bicarbonate. J Exp Mar Bio Ecol 199: 207–225.

[72] Brading P, Warner ME, Smith DJ, Suggett DJ (2013) Contrasting modes of inorganic carbon acquisition amongst *Symbiodinium* (Dinophyceae) phylotypes. New Phytol 200: 432–442. 23815769 10.1111/nph.12379

[73] Goiran C, Allemand D, Galgani I (1997) Transient Na^+^ stress in symbiotic dinoflagellates after isolation from coral-host cells and subsequent immersion in seawater. Mar Biol 129: 581–589.

[74] Seibt C, Schlichter D (2001) Compatible intracellular ion composition of the host improves carbon assimilation by zooxanthellae in mutualistic symbioses. Naturwissenschaften 88: 382–386. 11688413 10.1007/s001140100240

[75] Wang JT, Meng P-J, Sampayo EM, Tang SL, Chen CA (2011) Photosystem II breakdown induced by reactive oxygen species in freshly-isolated *Symbiodinium* from *Montipora* (Scleractinia; Acroporidae). Mar Ecol Prog Ser 422: 51–62.

[76] Sutton DC, Hoegh-Guldberg O (1990) Host-zooxanthella interactions in four temperate marine invertebrate symbioses: assessment of effect of host extracts on symbionts. Biol Bull 178: 175–186. 29314935 10.2307/1541975

