# Supplementary figures and images for: Symbiodinium Photosynthesis in Caribbean Octocorals
Source: PLoS One. 2014 Sep 5;9(9):e106419. doi: 10.1371/journal.pone.0106419 (PMC4156329; doi:10.1371/journal.pone.0106419)

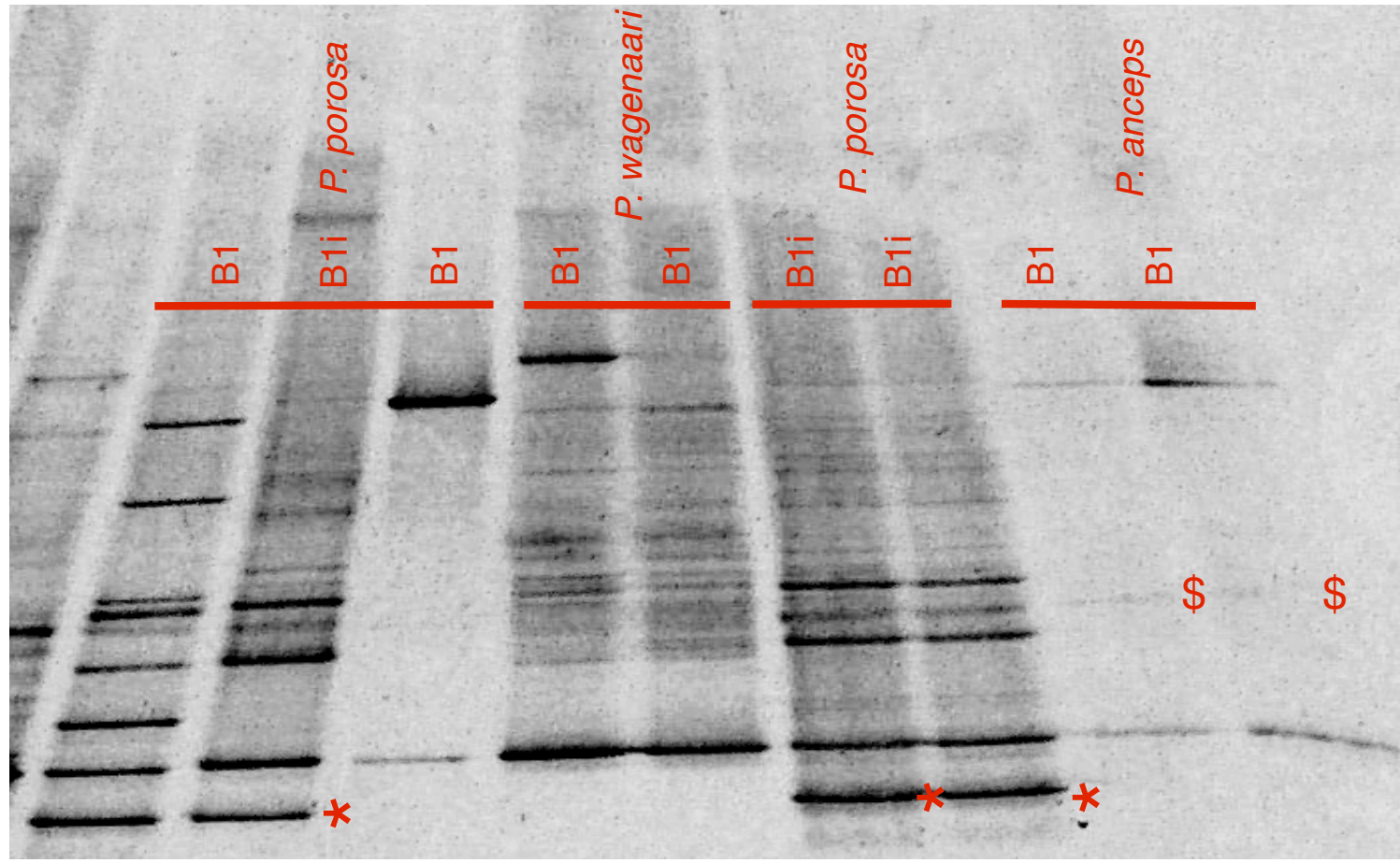

Supplement: Figure S1 — Denaturing gradient gel electrophoresis gel of ITS2 DNA from Symbiodinium associated with Pseudoplexaura porosa , Pseudoplexaura wagenaari , and Pterogorgia anceps . Type B1 Symbiodinium was recovered from all colonies of P. wagenaari and P. anceps, but from only 3 of 9 P. porosa colonies. B1 Symbiodinium exhibited distinct DGGE profiles. ($) denotes a band that is faint in these samples, but is typically visible in B1 Symbiodinium from P. anceps. (*) denotes the band of the type B1i ITS2 sequence variant. (PDF) [file pone.0106419.s001.pdf]
